# Fetal and Infant Effects of Maternal Opioid Use during Pregnancy: A Literature Review including Clinical, Toxicological, Pharmacogenomic, and Epigenetic Aspects for Forensic Evaluation

**DOI:** 10.3390/children11030278

**Published:** 2024-02-23

**Authors:** Elena Giovannini, Maria Paola Bonasoni, Jennifer Paola Pascali, Carla Bini, Guido Pelletti, Alberto Gualandi, Giovanni Dal Lago, Andrea Mercati, Beatrice Mariotti, Giulia Paola Pasini, Iarina Alexandra Poll, Paolo Fais

**Affiliations:** 1 Unit of Legal Medicine, Department of Medical and Surgical Sciences, University of Bologna, Via Irnerio 49, 40126 Bologna, Italy; elena.giovannini8@studio.unibo.it (E.G.); jennifer.pascali@unibo.it (J.P.P.); carla.bini@unibo.it (C.B.); guido.pelletti2@unibo.it (G.P.); giovanni.dallago3@studio.unibo.it (G.D.L.); andrea.mercati@studio.unibo.it (A.M.); beatrice.mariotti@studio.unibo.it (B.M.); giuliapaola.pasini@studio.unibo.it (G.P.P.); iarinaalexandra.poll@studio.unibo.it (I.A.P.); paolo.fais@unibo.it (P.F.); 2 Pathology Unit, Azienda USL-IRCCS di Reggio Emilia, Via Amendola 2, 42122 Reggio Emilia, Italy; 3 Institute of Pathology, DAME, University Hospital of Udine, 33100 Udine, Italy; a.gualandi@icloud.com

**Keywords:** opioids in pregnancy, methadone, buprenorphine, neonatal withdrawal symptoms from opioid drugs (NOWS), toxicology, pharmacogenomic, epigenetic

## Abstract

The two primary classes of opioid substances are morphine and its synthetic derivative, heroin. Opioids can cross the placental barrier, reaching fetal circulation. Therefore, at any gestational age, the fetus is highly exposed to pharmacologically active opioid metabolites and their associated adverse effects. This review aimed to investigate all the studies reported in a timeframe of forty years about prenatal and postnatal outcomes of opioid exposition during pregnancy. Clinical and toxicological aspects, as well as pharmacogenetic and epigenetic research focusing on fetal and infant effects of opioid use during pregnancy together with their medico-legal implications are exposed and discussed.

## 1. Introduction

Opioid dependence in pregnancy is a comprehensive biopsychosocial public health problem that raises many challenges for clinical and medico-legal specialists [1,2]. According to the American National Institute on Drug Abuse and the National Survey on Drug Use and Health, 5% to 8.3% of pregnant women use addictive substances, including opioids [3,4]. However, self-reporting by pregnant women of substance abuse or dependence often results in underestimation or misreporting. In particular, a diagnosis of substance abuse can have a significant impact on decisions about child custody, potentially resulting in a loss of parental responsibility [5,6].

The two primary classes of opioid substances are morphine and its synthetic derivative, heroin. They can cause severe acute side effects, such as respiratory depression, drowsiness, and impaired mental function. Chronic abuse can result in long-term effects on the brain’s white matter, ptentially affecting decision-making, behavioral control, and responses to stressful situations [7]. Opioid use disorder medications act on the same opioid receptors in the brain but do not produce the same effects. Methadone (MTD), classified as a full agonist opioid receptor, and buprenorphine, classified as a partial agonist opioid receptor, attach to and activate opioid receptors to relieve withdrawal symptoms and cravings [1,7]. Maintaining the use of opioid receptor agonists is also recommended for the treatment of heroin dependence during pregnancy [8,9,10].

Studies suggest that opioids can cross various surface barriers, including the placenta, and ultimately enter fetal circulation [11]. Consequently, at any gestational age, the fetus is exposed to clinically relevant concentrations of pharmacologically active opioid metabolites and their associated adverse effects [12,13,14].

In newborn infants, exposure to opioids during pregnancy often results in neonatal abstinence syndrome (NAS) [15]. NAS is a treatable collection of signs and symptoms observed in infants withdrawing from a variety of drugs, including heroin, MTD, amphetamine, methamphetamine, and benzodiazepines. It is most commonly associated with neonatal opioid withdrawal syndrome (NOWS). Approximately 75% of infants born to heroin-dependent mothers and almost all infants born to MTD-dependent mothers show withdrawal symptoms after birth [8,16,17].

The aim of this study is to provide a literature review, considering clinical and toxicological aspects as well as pharmacogenetic and epigenetic research focusing on fetal and infant effects of opioid use during pregnancy together with their medico-legal implications.

## 2. Materials and Methods

A literature search in the electronic databases PubMed, Scopus, and Web of Science was conducted using a combination of free text protocols as follows: (morphine OR heroin OR MTD OR buprenorphine OR opiod) AND (pregnancy OR fetus autopsy OR fetus death OR fetus postmortem OR stillbirth OR stillborn OR spontaneous abortion OR miscarriage OR neonatal death OR preterm delivery OR neonatal prematurity OR toxicology OR genetics OR epigenetics OR pharmacogenetics).

The English language and time interval of publication, from January 1970 to October 2023, were applied as filters. All studies that investigated the prenatal and postnatal outcomes of opioid exposition during pregnancy were included. Titles, abstracts, and full texts were screened for inclusion criteria and examined. References of the selected articles were further screened, and related papers were included as a source of additional data.

## 3. Results

**Figure 1:**
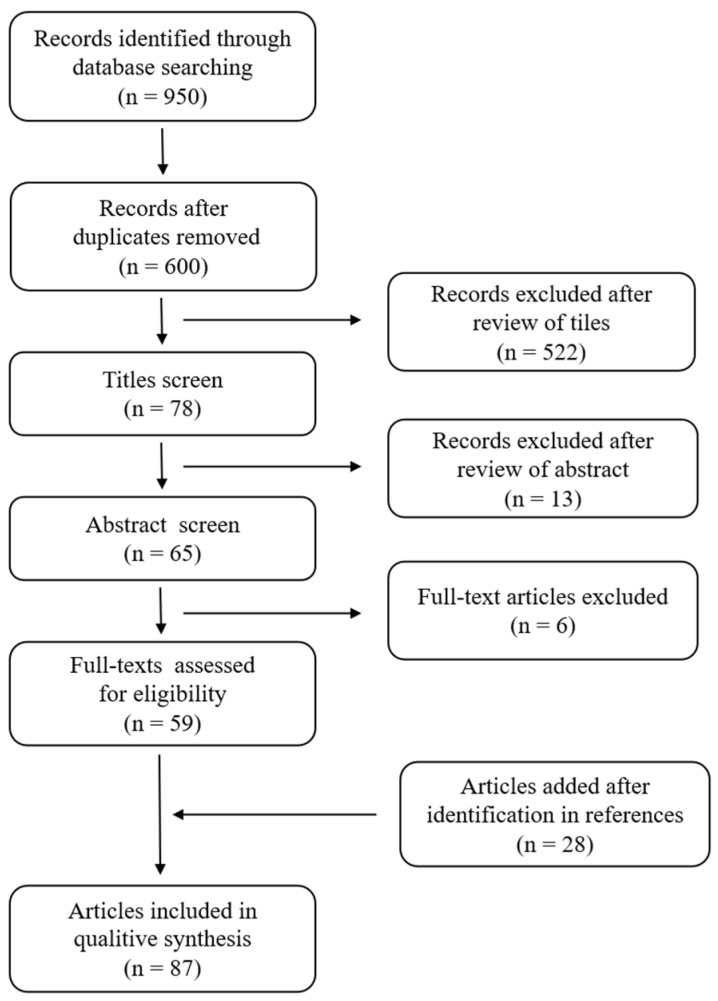
PRISMA flowchart of the systematic review.

**Figure 2:**
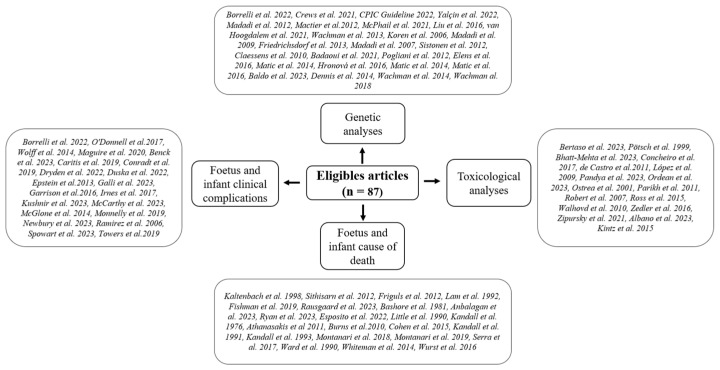
Studies organized by issue of interest. Foetus and infant clinical complications: Borrelli et al. 2022 [20], O’Donnell et al.2017 [22], Wolff et al. 2014 [24], Maguire et al. 2020 [26], Benck et al. 2023 [27], Caritis et al. 2019 [29], Conradt et al. 2019 [31], Dryden et al. 2022 [34], Duska et al. 2022 [35], Epstein et al.2013 [36], Galli et al. 2023 [37], Garrison et al.2016 [38], Irnes et al. 2017 [39], Kushnir et al. 2023 [42], McCarthy et al. 2023 [43], McGlone et al. 2014 [45], Monnelly et al. 2019 [46], Newbury et al. 2023 [49], Ramirez et al. 2006 [54], Spowart et al. 2023 [58], Towers et al.2019 [60]. Foetus and infant cause of death: Kaltenbach et al. 1998 [1], Sithisarn et al. 2012 [2], Friguls et al. 2012 [6], Lam et al. 1992 [9], Fishman et al. 2019 [11], Rausgaard et al. 2023 [14], Bashore et al. 1981 [16], Anbalagan et al. 2023 [17], Ryan et al. 2023 [18], Esposito et al. 2022 [19], Little et al. 1990 [21], Kandall et al. 1976 [23], Athanasakis et al 2011 [25], Burns et al.2010 [28], Cohen et al. 2015 [30], Kandall et al. 1991 [40], Kandall et al. 1993 [41], Montanari et al. 2018 [47], Montanari et al. 2019 [48], Serra et al. 2017 [57], Ward et al. 1990 [59], Whiteman et al. 2014 [61], Wurst et al. 2016 [63]. Genetic analyses: Borrelli et al. 2022 [20], Crews et al. 2021 [66], CPIC Guideline 2022 [67], Yalçin et al. 2022 [68], Madadi et al. 2012 [69], Mactier et al.2012 [70], McPhail et al. 2021 [71], Liu et al. 2016 [72], van Hoogdalem et al. 2021 [73], Wachman et al. 2013 [74], Koren et al. 2006 [77], Madadi et al. 2009 [78], Friedrichsdorf et al. 2013 [79], Madadi et al. 2007 [80], Sistonen et al. 2012 [81], Claessens et al. 2010 [82], Badaoui et al. 2021 [83], Pogliani et al. 2012 [84], Elens et al. 2016 [85], Matic et al. 2014 [86], Hronová et al. 2016 [87], Matic et al. 2014 [88], Matic et al. 2016 [89], Baldo et al. 2023 [90], Dennis et al. 2014 [91], Wachman et al. 2014 [92], Wachman al. 2018 [93]. Toxicological analyses: Bertaso et al. 2023 [5], Pötsch et al. 1999 [12], Bhatt-Mehta et al. 2023 [15], Concheiro et al. 2017 [32], de Castro et al.2011 [33], López et al. 2009 [44], Pandya et al. 2023 [50], Ordean et al. 2023 [51], Ostrea et al. 2001 [52], Parikh et al. 2011 [53], Robert et al. 2007 [55], Ross et al.2015 [56], Walhovd et al. 2010 [62], Zedler et al. 2016 [64], Zipursky et al. 2021 [65], Albano et al. 2023 [75], Kintz et al. 2015 [76].

The results of the literature search are summarized in Figure 1. A total of 87 studies met the inclusion criteria and were included in the review [1,2,5,6,9,11,12,14,15,16,17,18,19,20,21,22,23,24,25,26,27,28,29,30,31,32,33,34,35,36,37,38,39,40,41,42,43,44,45,46,47,48,49,50,51,52,53,54,55,56,57,58,59,60,61,62,63,64,65,66,67,68,69,70,71,72,73,74,75,76,77,78,79,80,81,82,83,84,85,86,87,88,89,90,91,92,93]. The papers were organized by issue of interest, as shown in Figure 2.

The specific details of each study are provided in the Supplementary Tables (Tables S1–S4).

## 4. Discussion

### 4.1. Clinical Implications for the Fetus and Infant

#### 4.1.1. Pregnancy Complications

During pregnancy, exposure to opioids can lead to complications in both the fetus and the fetal adnexa.

Intrauterine growth retardation and restriction frequently accompany heroin in utero exposure because the oxygen demands of the developing fetus may not be met, especially as gestation progresses. Significant reductions in cell number have been observed in many organs, particularly in brain size [1,8,17,23]. Congenital anomalies have been reported with opioid use in pregnancy, especially cardiovascular defects (conoventricular, atrial, ventricular, atrioventricular) and hypoplastic left heart syndrome. Spina bifida, clubfoot, and oro-facial clefts also seem to be the most frequent related malformations [18].

Prescription opioids may modestly increase the risk of placental abruption as a result of a cumulative exposure effect either in early or late stages, likely reflecting a condition of chronic placental insufficiency and placental bed ischemia. However, opioids do not seem to be associated with the development of preeclampsia in addicted mothers; maternal hemorrhage from placenta previa and abruptio placentae is four times higher than in the normal population, and preeclampsia is even three times more frequent [19]. Opioid addiction in pregnancy may complicate the management of labor pains, as the women develop tolerance [16].

Concerning childbirth, the consumption of heroin, in particular, increases the likelihood of preterm delivery and, consequently, preterm birth [17,19,21]. Moreover, premature rupture of the membrane is twice as common in addicts and can lead to severe complications, such as intrauterine infection and neonatal sepsis [16].

In the antepartum period, fetal heart rate (FHR) monitoring helps to assess fetal health. Heart rate baseline variability and heart rate accelerations represent the basic fetal cardiovascular reflexes produced as a rection to fetal movements. An abnormal FHR may underlie a fetal hypoxic status, but in addicted women, MTD and heroin weaken fetal cardiovascular reflexes due to their sedative effects, and FHR may be unreliable [16].

In pregnancy, MTD maintenance therapy is related to a delay in FHR accelerations in nonstress tests. In a recent study, the correlation between MTD maintenance therapy and FHR patterns was examined during labor, after maintaining a constant dose of MTD four weeks before delivery. During the first stage of labor, FHR was detected altered with reduced variability, a diminished number of accelerations, and a lower baseline. However, these abnormal FHR patterns were not related to fetal acidosis or dose administered. In the second stage of labor, more FHR decelerations were recorded in the fetuses of mothers undergoing MTD maintenance therapy. The necessity for operative delivery was similar to mothers not exposed, irrespective of dose, except repetitive late or severe variable decelerations were more worrisome in the second stage. No clear reason was found to comprehend the more frequent decelerations in late labor, as the newborns’ Apgar score was within normal limits.

In drug-addicted women, meconium staining of the amniotic fluid is three times higher, probably due to fetal hypoxia before or during labor [16].

Complications during pregnancy in women dependent on opioids include maternal conditions such as chorioamnionitis, postpartum hemorrhage, sepsis, and thrombophlebitis. The incidence of these complications is lower in women maintained on MTD than in those using heroin [1]. In general, pregnant women addicted to heroin experience a decline in health status, which affects both fetal development and the ability to care for the child after birth. Common problems include malnutrition, anemia, and inadequate maternal weight gain, resulting in low birth weight and increased perinatal mortality [16]. The obstetric management of opioid-dependent pregnant women is challenging because of the multiple medical complications resulting from chronic intravenous opiate abuse. Infections, particularly hepatitis A, B, and C; tuberculosis; bacterial endocarditis; septicemia; cellulitis; and sexually transmitted diseases, are major contributors to these complications [1,94,95]. In addition, the care of opioid-dependent women at risk of complications in the peripartum period is particularly challenging. Chronic opioid use and the development of tolerance may require high doses of analgesia, leading to persistent pain during labor [22,96,97,98].

#### 4.1.2. Postnatal Complications

##### Neonatal Abstinence Syndrome: Neonatal Opioid Withdrawal Syndrome (NOWS)

NOWS can present with a variety of signs and symptoms affecting different organs. Neurological symptoms commonly described include increased alertness, irritability, hyperreflexia, hypertonicity, and sleep disturbance. Symptoms of autonomic hyperreactivity such as increased sweating, yawning, and sneezing are commonly reported. Other manifestations include uncoordinated and constant sucking, poor feeding, inadequate weight gain, vomiting, diarrhea, and dehydration. Various factors influence the incidence and severity of withdrawal in individual infants, including the type of substances used by the mother, the timing and dosage before delivery, the nature of the labor, anesthesia or analgesia during labor, and the maturity, nutritional status, and general health of the infant. Withdrawal may have different characteristics, ranging from mild and transient to delayed or progressive. In some cases, it may be intermittent or have a biphasic course with acute neonatal withdrawal signs followed by improvement and then the onset of subacute withdrawal [1,8,9,16,17,65,99].

The majority of cases of NOWS resolve within seven days with appropriate treatment [15,65]. Non-pharmacological management, adapted to the symptoms of the newborn, may include providing a low-stimulation environment, with gentle handling and soothing techniques such as breastfeeding, swaddling, and skin-to-skin contact [99]. In severe cases, opioid treatment with morphine or MTD may be required. Early identification of neonates with NOWS requiring pharmacological intervention may reduce drug exposure, treatment duration, and hospital admissions [20].

Withdrawal symptoms may occur within hours to two weeks after birth, with most symptoms occurring within 72 h. The shorter half-life of heroin results in earlier withdrawal symptoms compared with MTD-exposed infants. The duration of NOWS may be longer with MTD, and symptoms may not appear for 24–72 h due to the extended half-life of MTD of approximately 24 h. The duration of symptoms varies and is influenced by gestational age and the infant’s metabolic and excretory pathways [8].

Behavioral treatment, MTD pharmacotherapy, and appropriate prenatal care reduce obstetric and fetal complications and neonatal morbidity and mortality. However, there is still debate about MTD withdrawal during pregnancy, appropriate doses, gestational dose adjustments, and the relationship between maternal MTD dose and NOWS severity. Pregnant women with a history of substance dependence are recommended to maintain the lowest MTD dose for stability and are encouraged to breastfeed, as breastfeeding helps to calm fussy infants and may alleviate withdrawal symptoms. The benefits of breastfeeding extend to preterm infants who are unable to suckle [34].

Neonates exposed to buprenorphine were less likely to require NOWS treatment compared with MTD-exposed neonates, according to a recent study [51]. However, among neonates requiring treatment, there was no significant difference in severity, duration of medical treatment, or length of stay between MTD and buprenorphine exposure. Thus, the treatment of pregnant women with buprenorphine reduces the risk of developing NOWS, but the type of maternal prenatal exposure becomes irrelevant once treatment is initiated [42].

##### Complications Due to Direct Opioid Exposure

Heroin-exposed infants exhibit low birth weight and head circumference at birth, not only in cases of prematurity but also when gestational age is normal [8,17,38,60,61]. It has been observed that past heroin abuse, despite abstinence during a current pregnancy, may still contribute to fetal growth retardation and low birth weight [23,40,41].

Women in MTD maintenance therapy also have an increased risk of low birth weight and preterm birth, although this is significantly lower than for babies whose mothers abused heroin without MTD maintenance therapy [34,100]. This suggests that MTD administration to opiate-addicted mothers promotes fetal growth in a dose-related manner [8]. When comparing the effects of MTD with buprenorphine, a lower risk of preterm birth, greater birth weight, and larger head circumference were observed with MTD use. No differences were noted for congenital anomalies and other fetal growth measures [64].

Heroin exposure is also associated with systemic complications affecting multiple organs, such as neonatal asphyxia, intracranial hemorrhage, hypoglycemia, hypocalcemia, septicemia, and hyperbilirubinemia [8,17].

MTD exposure can elevate the cardiovascular risk of cardiac septal defects and congenital long QT syndrome or other arrhythmias [43,53]. Additionally, rat experiments have shown that prenatal morphine exposure increases the risk of hypertension in adult life, with the increase in mean arterial pressure correlating with higher levels of in vivo angiotensin II, inducing vasoconstriction [101].

Significant differences in developmental outcomes have been reported in infants exposed to opioids [31]. Infants affected by NOWS often have visual–motor problems such as strabismus and nystagmus, as well as impaired visual acuity due to refractive errors. Children with prenatal opioid exposure show notable cognitive deficits, lower verbal performance, impaired short-term memory, and impaired executive function compared to controls over the age of three. Academic test scores of NOWS children show consistently lower mean scores in all grades and subjects, with the greatest disparity observed in grade seven. Prenatal opioid exposure is associated with psycho-behavioral problems, including attention-deficit/hyperactivity disorder, conduct disorder, and anxiety disorder [17,102]. Radiological analysis indicates reduced regional cerebral volumes, including the cerebral cortex, amygdala, accumbens, putamen, pallidum, brainstem, and cerebellum, in children exposed to opioids in utero. Smaller volumes are seen in the basal ganglia, thalamus, and cerebellar white matter compared with matched unexposed controls [39,62].

In addition, children born to mothers treated with MTD or buprenorphine during pregnancy have significantly worse neurodevelopmental outcomes, including cognitive, psychomotor, and executive functioning; observed motor activity, behavior, and attention; and long-term visual outcomes, including congenital nystagmus, refractive errors, visual acuity, and contrast sensitivity, compared with children born to non-users [26,37,45,46,58]. A recent study suggests that buprenorphine use during pregnancy is associated with a lower risk of adverse outcomes than MTD use, particularly in relation to indices of behavioral, emotional, and cognitive regulation [43].

### 4.2. Fetus and Infant Cause of Death

#### 4.2.1. Intrauterine Fetal Death

Opioid abuse in pregnancy exposes the fetus to multiple episodes of intoxication and withdrawal [8]. Intrauterine fetal death (IUFD) may occur during the withdrawal period, as fetal movements tend to increase followed by stillness. Intensified fetal psychomotor activity determines a higher oxygen demand, and if not adequately supplied, it may result in stillbirth until the IUFD [16].

As the stillbirth risk is higher in intravenous opioid-abusing women, weekly nonstress tests should be carried out starting from the 32nd week of gestation. In women undergoing MTD maintenance therapy, no increased incidence of IUFD has been reported. Total opioid abstinence is recommended in pregnancy, but acute withdrawal must be avoided, as it induces fetal distress, possibly leading to intrauterine growth restriction, preterm labor and birth, and IUFD [64].

MTD maintenance therapy is generally safe in pregnancy, but MTD can be associated with placental delayed villous maturation (DVM) and therefore potential placental insufficiency. DVM features include irregular and plump villi and reduced vasculo-syncytial membranes. The latter impairs fetal–maternal blood exchange, hampering fetal well-being [57].

A recent case concerned a stillborn infant whose mother was receiving MTD maintenance therapy. The reported cause of death was identified as a fatal hypoxic–ischemic condition resulting from a combination of chronic exposure to MTD during fetal development, widespread placental vascular abnormalities, and partial non-occlusive thrombosis in both the umbilical vein and its chorionic branches [47]. The IUFD occurred at 39 weeks of gestation, and the baby presented intrauterine growth restriction with asymmetrical growth and lower anthropometrical parameters (weight, head circumference, crown heel length). Postmortem external examination showed evidence of acute hypoxia with no maceration but the presence of lip cyanosis and conjunctival petechiae. Internal examination disclosed abundant pleural and pericardial petechiae but no anatomical anomalies. The brain presented edema with flattened sulci and circumvolutions, a pale cortex, and a brownish white matter, consistent with “ribbon effect” features. Histology confirmed cerebral hypoxic changes showing red neurons, glial karyorrhexis, and plump endothelial cells. However, reactive ramified astrocytes and mild reactive subcortical gliosis might have reflected a chronic non-specific injury. The other fetal tissues were well preserved with minimal autolysis. Placental examination confirmed the gross findings of thrombosis in the umbilical vein and its chorionic ramifications. Histologically, DVM was widespread, with focal clusters of avascular villi, a result of compromised cord and chorionic blood circulation. Moreover, toxicological analysis revealed a high concentration of MTD and its metabolite EDDP (2-ethylidene-1,5-dimethyl-3,3-diphenylpyrrolidine) in the liver (2200 and 5720 ng/g) and kidneys (15,000 and 31,300 ng/g). In neonates and children, the clearance of MTD is reduced due to enzyme immaturity. However, as it has never been studied in fetuses, the underdevelopment of detoxification enzymes and slow glomerular filtration may similarly prolong MTD half-life, thus prolonging substance accumulation and fetal intoxication. A thorough description of only one case, however, is merely suggestive of this hypothesis. The toxicological results of this case are better discussed in the relevant paragraph.

#### 4.2.2. Infant Death

Neonatal mortality is four times higher among drug users compared to the general population. It is crucial to differentiate between cases where the underlying causes of death are identified and cases where the cause of death remains unknown, resulting in a classification as sudden infant death syndrome (SIDS), recently termed sudden unexpected death in infancy (SUDI). SUDI is defined as the sudden, unexpected death of an infant from birth to 12 months of age that is not anticipated 24 h before, in the absence of a known pre-existing medical cause of death [93].

The primary causes of infant death include infectious and systemic complications associated with newborn prematurity and growth retardation, particularly when these conditions coexist [16]. Additionally, infant death may occur in cases of severe and inadequately treated NOWS, especially when excessively protracted [8].

When the cause of death is not clearly identified, these cases are classified under the broader clinical term SIDS. SIDS is characterized by the sudden death of an infant under one year of age that remains unexplained after a full investigation, including a thorough autopsy, analysis of the death scene, and review of the clinical history [8,30,40,41,103,104]. The likelihood of sudden death increases 2–5 times in neonates whose mothers have used opiates. It has been hypothesized that this is due to the direct toxicity of these substances and their interaction with congenital risk factors, such as genetic mutations [25,105].

There is increasing evidence of a possible link between SIDS and long QT syndrome, which leads to arrhythmias and sudden death in newborns [30,106].

Studies suggest, in particular, that infants born to mothers undergoing MTD treatment may experience QTc prolongation. MTD treatment is known to cause QTc prolongation in adults, leading to torsade de pointes. Transient and considered benign, QTc prolongation of up to 500 ms can occur in apparently healthy newborns. The maternal use of MTD is associated with an increased QTc interval in newborns during the first two days of life compared with unexposed infants. Therefore, it is recommended as a precautionary measure to investigate any family history of syncope or sudden death in mothers receiving MTD. In addition, any heart rate abnormalities, such as bradycardia or tachycardia, in infants born to these mothers should prompt further investigation, including a 12-lead electrocardiogram [53].

### 4.3. Toxicological Analyses

Toxicological analyses on samples from newborns face no specific analytical problem, excepting the lower amounts of specimens versus adults. However, the same procedures can be used for sample pretreatment and instrumental analyses. The main issue is the interpretation of the results, in reason of the different pharmacological parameters due to incomplete organ development. For example, kidney activity is limited in the fetal stage, with a renal blood flow of only 3% of the cardiac output compared with 25% in the adult stage, and, as a consequence, urine is not the main route of drug elimination. The capacity of fetal hepatic metabolism depends on fetal maturation, with CYP-3A4 and CYP-2D6 being present by week 20, but the lack of other important enzymes hampers the ability of complete detoxification. Low fetal drug-metabolizing capacity and minimal drug elimination from the amniotic fluid by slow exchange in advancing pregnancy play a crucial role in xenobiotic toxicity. On this basis, xenobiotics such as heroin and its pharmacologically active metabolites, 6-monoacetyl -morphine and morphine, can persist in the fetal unit, and especially in the central nervous system, for much longer times than in maternal circulation [12]. The impact on the developing nervous system varies from its effects on mature systems, especially before homeostatic regulatory mechanisms are adequately calibrated. Unique to the pregnancy stage is the placenta, which is a transient organ separating maternal and fetal blood circulation. It is permeable to the vast majority of drugs of abuse and can be a target for drug toxicity or metabolism itself [56].

For example, MTD crosses the human placenta in different ways: metabolism, simple diffusion, and carrier-mediated uptake/efflux; it is also retained by the placenta, leading to a 5-fold less concentration in cord blood. Placental metabolism is responsible for MTD transfer to the fetus, although inactivation by placenta enzymes is limited. In the case of a stillborn baby delivered at 39 weeks of gestation by a mother under an 80 mg/day treatment, MTD and its main metabolite EDDP were quantified in brain, liver, and kidney tissues via ultra-high-performance liquid chromatography coupled to mass spectrometry (UHPLC-MS/MS). High levels of MTD and EDDP were detected in all samples. In particular, the highest concentrations of MTD and EDDP were observed in the kidneys, probably because of their small size and their undeveloped glomerular filtration activity, which might have resulted in poor excretion. Liver accumulation was also observed, probably due to its small size and reduced detoxification capacity. The organ with the lowest levels was the brain. Similar EDDP/methadone ratios were observed in the liver and kidneys, but in the brain, MTD was predominant. Difficulties of EDDP passing through the blood–brain barrier may be the explanation of the inverted EDDP/MTD ratio observed in the brain [47].

The selection of the appropriate sample to analyze to detect opioid exposure in utero depends on sample availability and specific clinical or forensic considerations [75].

Meconium is the preferred matrix for in utero drug testing due to its easily collectible and non-invasive nature, offering a broader window for drug detection compared to urine. It primarily reflects prenatal exposure from the third trimester of pregnancy. However, meconium collection can be challenging in certain situations, such as delayed expulsion in premature infants or early discharge in cases of fetal distress before delivery. The umbilical cord has been proposed as an alternative to meconium, given its immediate availability postdelivery. Methadone and EDDP levels in the umbilical cord have demonstrated a correlation with maternal methadone dosage and neonatal outcomes, also serving as predictors of Neonatal Opioid Withdrawal Syndrome (NOWS) severity. Nevertheless, meconium has proven more adept at detecting a greater number of in utero exposures to cocaine and opiates, thanks to its extended detection window [33].

At the moment, despite the fact that meconium is the preferred specimen to detect neonatal drug exposure, the analysis of this specimen requires specialized laboratories. Nevertheless, drug deposition and detection in meconium are contingent on various factors. These include the timing and frequency of drug use, maternal and fetal metabolism, urine contamination, placental transfer, and pre-analytical factors such as specimen quality and storage conditions. Testing for in utero drug exposure in meconium specimens should be performed with the most sensitive analytical techniques, such as gas or liquid chromatography coupled to mass spectrometry (GC-MS or LC-MS) since the concentrations may be very low, and, thus, the traditional immunological screening used for toxicological analysis, which are calibrated at higher cut-offs, may produce initial false negative results [50]. Urine and meconium could be used as complementary matrices for the evaluation of exposure during pregnancy. A newborn presenting symptoms of hyperexcitability was submitted to toxicological analysis for opiates via urine and meconium tests. An ad hoc GC-MS method had been developed on the meconium, and the results were positive for both matrices, demonstrating exposure to opiates during the last 20 weeks of gestation for meconium and a few hours prior to birth in the case of urinalysis [44]. Meconium can also test positive for morphine and metabolites if this drug is administered during labor and delivery [32].

In forensic toxicology, hair analysis is required to complement blood and/or urine analyses since it allows for differentiation between single exposure and chronic use of a drug; hair samples can also be analyzed by segmentation to obtain information on drug exposure for a stated period. However, differences between the hair of children and adults exist; thickness, porosity, and the ratio of anagen/catagen phases are different, and the growth rate can contrast with the usual 1 cm/month considered normal for adults. For these reasons, any interpretation on hair toxicological findings in children should be taken with extreme care. In the case of newborns, hair can provide data on in utero exposure to xenobiotics derived from the mother’s consumption habits. During fetal development, hair morphology changes; activation of the hair follicles starts at about the 4th month of pregnancy, producing the lanugo. The lanugo is replaced by downy, short, non-pigmented, thin hair, and starting from the 8th month of pregnancy, the final hair appears. In all these phases, hair growth varies greatly. Therefore, it is very difficult to establish a window of detection when testing for drugs in young children. Interpretation is even more complicated, as drugs incorporated in the hair of the fetus during pregnancy contribute to positive findings for a time window after delivery, and no data are available in the literature on the disappearance interval after delivery [76].

In a recent study, buprenorphine was analyzed in the hair of four mother–newborn dyads. The mothers were under treatment with buprenorphine during the third trimester. All the hair from the newborns had hair specimens positive for buprenorphine and norbuprenorphine. The results also showed a significant positive association between cumulative maternal buprenorphine doses and total concentrations of buprenorphine and its metabolite in maternal hair, while no correlation between cumulative maternal doses and buprenorphine/metabolite in newborn hair concentrations was found [55]. Hair testing has proven to be more effective in detecting prenatal exposure to opiates during the third trimester than urine and meconium, providing a higher number of positive results; in fact, 6-MAM could be detected and determined in hair more frequently, and this is important since 6-MAM is the only specific heroin metabolite suitable for confirming heroin use. Neonatal hair can supply information when other biological matrices are not available anymore or insufficient for analysis. Besides neonatal hair, maternal hair can also be used to detect the perinatal use of opioids. The main limitation of maternal hair analysis is its susceptibility to false positive test results, particularly linked to passive exposure to drugs of abuse. To avoid any erroneous interpretation of the results due to involuntary contamination of the hair, three points should be addressed. Firstly, external decontamination of hair must be performed before the analysis. Subsequently, the detection of parent compound and metabolites combined with the appropriate cut-off levels that represent decisional levels near the limit of quantification of the analytical technique must be carried out. In relation to its growth (1 cm/month), hair analysis offers a wider window of detection than urine and blood and, through segmental analysis, can identify the time of use/abuse in mothers. On this basis, maternal hair testing should be used to detect illicit drug use during pregnancy in suspect cases or whenever neonatal withdrawal syndrome is observed [32].

### 4.4. The Pharmacogenetics of Opioids in Pregnancy and Epigenetics Research

#### 4.4.1. Pharmacogenomic of Opioids in Pregnancy: Fetal and Infant Effects

In neonates, pharmacogenetics introduces an extra level of complexity beyond genetic variability, involving polymorphisms in genes associated with drug pharmacokinetics (PK) (such as metabolism and transporters) or pharmacodynamics (PD) (such as receptors and target enzymes).

The rapid evolution of ontogenetic changes in the expression of these proteins or processes is linked to the maturation of renal, cardiac, intestinal, and other physiological aspects, leading to a maturational, age-related mismatch between genotype and phenotype, defined as “phenoconversion” [68].

Despite similar maternal exposures, genetic factors may be responsible for this significant interpatient variation in the NOWS phenotype. Variations in single nucleotide polymorphisms (SNPs) and/or other sequence variants, including insertions and deletions within crucial candidate genes, have been recognized as significant factors affecting the risk of opioid addiction and acting as moderators of responses to opioid therapy in adults [91], but there is still a significant knowledge gap regarding the PK/PD of NOWS pharmacotherapies in infants.

In NOWS, morphine, MTD, and buprenorphine are commonly employed as primary treatments, while clonidine and phenobarbital are generally reserved for supplementary therapy.

The PK/PD approach has been employed in NOWS pharmacotherapies to study interpatient variability in drug disposition and action, the exposure–response relationship, and the optimization of dosing protocols [72,107,108].

Gestational age significantly influences altered PK. This is demonstrated by a diminished clearance of morphine, MTD, and, to a lesser extent, phenobarbital in preterm infants. Moreover, the postnatal maturation of enzymatic and physiological functions has a substantial impact on the PK and disposition of numerous drugs employed in the treatment of NOWS [73].

Conclusive findings regarding the correlation (or lack) between genetic variations in *CYP2D6*, *CYP2B6*, *CYP3A*, *ABCB1*, *OPRM1*, *OCT1*, and/or *COMT* genes and the response to opioids have been reported in many studies.

##### CYP2D6

Codeine, clonidine, tramadol, hydrocodone, oxycodone, and methadone are some of the opioids that are metabolized by *CYP2D6* to some extent.

The principal sources of analgesia and respiratory depression originate from codeine transformation into morphine in the liver, primarily facilitated by *CYP2D6*, and its activity varies among individuals due to genetic polymorphisms. The moderate increase in opioid-related adverse events, including fatal toxicities, in infants breastfed by *CYP2D6* ultrarapid-metabolizing mothers proved it [77,78,79,80,81].

Clonidine, an antihypertensive agent, is presently reserved for adjunctive therapy in the treatment of NOWS. The PK/PD relationship of clonidine in NOWS has not yet been established. Approximately 60% of the clonidine dose is eliminated unchanged through renal excretion in adults; the remaining portion undergoes hepatic metabolism, predominantly through CYP2D6-catalyzed 4-hydroxylation, resulting in the formation of 4-hydroxyclonidine (COH) [82,109].

The rate of clearance for clonidine is lower in newborns than in adults, peaking at 50% within two months and 90% at one year postnatal age [101]. The maturation of *CYP2D6* may be involved, as the enzyme’s activity in the first week of life decreases, particularly in premature infants [110].

The impact of the *CYP2D6* phenotype and gestational age on fetal morphine exposure following codeine administration to 250 pregnant females was investigated using an informed precision dosing model [83]. A clinically significant (>1.65-fold) increase in the fetal morphine concentration time curve (AUC) was observed in the *CYP2D6* UM phenotype cohort when compared to the *CYP2D6* extensive metabolizer (EM) and poor metabolizer (PM) phenotype cohorts at all gestational stages. The fetal morphine AUC was 1.5- to 1.75-fold higher during the initial trimester of the *CYP2D6* EM and UM cohorts, despite the rise in morphine formation associated with increased *CYP2D6* expression during pregnancy. These findings imply that the highest risk concerning fetal morphine exposure may occur during the initial trimester of pregnancy.

##### OPRM1, COMT, and ABCB1

Other genes that have been studied for their association with opioid clinical effects or adverse events include *OPRM1* (μ-opioid receptor), *COMT* (catechol-o-methyltransferase), and *ABCB1* (multidrug resistance). Among infants with NOWS, variants in the *OPRM1* and *COMT* genes were associated with a shorter length in hospital stay and less need of treatment. The OPRM1 118A>G AG/GG genotypes have shorter hospital stays for treatment of NOWS than the AA genotype, and mothers with the rs1799971 AG or GG genotype have infants who are less likely to require treatment for NOWS (buprenorphine, MTD), but association with the *ABCB1* SNPs and the likelihood of withdrawal syndrome was not significant [74]. Moreover, a single case study documented a temporary occurrence of bilateral hydronephrosis and acute kidney injury caused by a bladder sphincter spasm in a preterm newborn (GA 34 weeks); the pharmacogenomic screening revealed that the patient was homozygous for the C3435T polymorphism in the *ABCB1* gene, linked to diminished P-glycoprotein activity. Given that P-glycoprotein is also expressed in urothelial cells, the authors proposed that this polymorphism played a role in opioid-induced urinary retention [84].

Elens et al. concluded that the response of preterm infants to opioid premedication for intubation is associated with the KCNJ6 –1250G>A and the COMT c.472G>A (rs4680, Val158Met) polymorphisms. The presence of the KCNJ6 -1250A allele and the COMT158Val allele appears to be predispose to diminished opioid-induced pain relief [85]. The potential association between SNPs of OPRM1 118A>G (asn40asp), COMT 472G>A (val158met), and beta-arrestin-2 (G-protein coupled receptor, ARRB2) 8622C>T and the requirement for morphine rescue was evaluated by Matic et al., but neither *OPRM1* nor *COMT* SNPs showed a statistically significant increase in the risk of rescue morphine or the morphine dose [106]. However, individuals with the combined *OPRM1/COMT* “high-risk” genotype exhibited a higher likelihood of requiring rescue (Odds Ratio: 5.12).

Hronova et al. investigated the influence of *COMT* and the human pregnant X receptor (PXR), in addition to ABCB1, on sufentanil and midazolam dosing, showing that these polymorphisms had an impact on dosing but did not affect the depth of analgo-sedation or the occurrence of withdrawal syndrome [87].

##### UDP-Glucoronosyl-Transferase (UGT) and Organic Cation Transporter 1 (OCT1)

For UDP-Glucoronosyl-Transferase (UGT), morphine plasma concentrations in 17 preterm neonates, with a gestational age range from 25 to 32 weeks, were associated with postnatal age and UGT2B7 −900G>A, while the morphine-3 and morphine-6-glucuronide/morphine ratio was higher compared to the −900G/G type, indicating that morphine metabolism in preterm infants is significantly altered by the UGT2B7 -900G>A polymorphism [88].

The impact of genetic variations at the organic cation transporter 1 (OCT1) and UDP-glucuronosyltransferase 2B7 (UGT2B7) loci on age-dependent morphine clearance was assessed in a cohort of critically ill neonatal patients by Hahn D et al., finding a notable influence of the *OCT1* genotype and gestational age on morphine pharmacokinetics (PKs) [107]. Indeed, the *OCT1* genotype influences morphine clearance in term and post-term neonates, suggesting that knowledge of the OCT1 genotype could help identify patients at risk for more severe effects due to lower morphine clearance.

Matic et al. reported evidence that *OCT1* activity in infancy is susceptible to genetic variability, as the *SLC22A1/OCT1* genotype associated with the number of *CYP2D6* functional gene copies influenced the O-desmethyltramadol disposition, indicating a rapid maturation of *OCT1* after birth, in both term and preterm infants [89].

Related to Multidrug Resistance Protein 3 (MRP3) polymorphisms, a decreasing trend in morphine clearance was observed with MRP3 efflux transporter (rs4793665) genotypes (CC>CT>TT) in a subgroup of OCT1 wild-type cases [111].

##### Methadone Pharmacogenetics and CYP2B6

NOWS in infants of methadone-maintained opioid-dependent mothers was investigated, and pharmacogenetic analysis of candidate genes was conducted to identify genetic variants that might have made infants susceptible to either MTD accumulation or heightened sensitivity, as part of the investigations of death [16,21,48,70,78,90,94,95]. Methadone metabolism can be modified by polymorphic variations of *CYP2B6* and *ABCB1*. The most popular gene studied in cases of methadone intoxication is *CYP2B6*, both in healthy patients [112,113,114,115] and in fatalities [78,116,117]. The primary route of methadone degradation is through the N-demethylation of methadone, which results in an inactive EDDP. Variations in NOWS infant responses to methadone are associated with SNPs in *CYP2B6*, and those in the *OPRM1*, *ABCB1*, and *COMT* genes are associated with hospital stay length.

In Mactier et al., there were no differences observed between treated and untreated neonates concerning *ABCB1*, *COMT*, and *CYP2D6*. Regarding *CYP2B6*, the homozygous (wild type) alleles CYP2B6 516 G > T and 785 A > G are associated with normal enzyme function and are carried by 75% of the Caucasian population [70]. However, the genomic variation in CYP2B6 between treated and untreated neonates with methadone was significant, as untreated infants were much more likely to carry alleles associated with decreased enzyme function at CYP2B6 516 G>T and 785 A>G. Similar findings were observed in the meta-analysis performed by Dennis et al., suggesting that MTD metabolism is significantly slower in *6 homozygous carriers and concluding that the severity of NOWS is related to the decline of the neonate’s plasma MTD concentration over the first four postnatal days [91].

In a case study of elevated MTD findings in deceased neonates who were exclusively breastfed by mothers enrolled in MTD maintenance programs, a pharmacogenetic analysis was conducted to examine variants linked to methadone metabolism and response [78]. A male infant was found to be homozygous for the CYP2B6*6 haplotype, a genetic pattern linked to MTD-related mortality in adults due to compromised MTD metabolism, and, additionally, he was also heterozygous for SNPs in the *ABCB1* gene, which are associated with reduced efflux activity and with the potential to make an individual more susceptible to the opioid-mediated effects of MTD. A postmortem examination did not uncover any pathological causes to elucidate the cause of death, which remained undetermined, assuming polygenic factors as having the potential to increase the vulnerability of specific infants to the effects of methadone.

In a stillborn female fetus affected by chronic methadone intoxication, four *CYP2B6* common SNPs (rs3745274, rs2279343, rs3211371, rs8192719), five *ABCB1* common SNPs (rs2032582, rs1045642, rs1128503, rs2229109, rs9282564), and one *OPRM1* SNP (rs1799971) were genotyped to investigate the correlation between the maternal consumption of MTD, the accumulation of the drug in fetal tissues, and the resulting fatality of the fetus linked to intoxication. A comprehensive pattern emerged from genetic studies related to the stillborn death linked to methadone intoxication. The fetus was found to be homozygous for variant alleles in *CYP2B6* SNPs (rs3745274TT, rs2279343GG, rs8192719TT) and heterozygous for rs3211371, providing a genetic explanation for the notable drug accumulation observed in the fetal tissues as probably determined by the slow MTD metabolizer phenotype. The stillborn was also homozygous for wild-type alleles in all typed SNPs in *ABCB1* [rs1128503 (GG), rs2032582 (CC), rs1045642 (GG), rs2229109 (GG), and rs9282564 (TT) and for the wild-type allele rs1799971 in *OPRM1*. The stillborn could not have had a consistent metabolism of methadone and consistent excretion, given the expected outcome of premature death [47].

#### 4.4.2. Epigenetics

Epigenetic modifications, such as DNA methylation and chromatin remodeling, are induced by various factors like environmental conditions, age, and substance abuse, including opioids [118,119]. These modifications impact the function of encoded proteins without altering the DNA sequence and can influence the severity of diseases associated with maternal and fetal drug metabolism. In a study aimed at exploring potential correlations between DNA methylation levels in OPRM1 promoters in opioid-exposed neonates and the outcomes of withdrawal symptoms, the assessment of hypermethylation in the OPRM1 promoter showed that elevated methylation was linked to more unfavorable treatment outcomes [92]. Elevated methylation levels in the infants were correlated with an increased requirement for pharmacologic treatment; in mothers, higher levels of methylation were linked to an extended hospital stay for their infants [93].

In a different epigenetic investigation, the DNA methylation status of *ABCB1*, *CYP2D6*, and *OPRM1* genes in opioid-dependent mothers and their infants indicated that opioid-exposed newborns and mothers exhibited comparable DNA methylation for *ABCB1* and *CYP2D6*. There was less methylation for *OPRM1* and greater methylation for *ABCB1*. The authors were uncertain whether these findings were attributable to opioid exposure or other factors [70].

Finally, epigenetic modifications in the placenta caused by prenatal opioid exposure may lead to placental dysfunction, resulting in abnormal fetal brain development and the manifestation of opioid withdrawal symptoms in neonates as described by [20].

## 5. Conclusions

This review highlights the fact that prenatal exposure to opioids can result in the development of various complications during pregnancy and post-birth, potentially leading to serious consequences, including fetal death. While clinical complications arising from withdrawal syndrome and direct organ damage are well documented, the underlying pathophysiological processes causing intrauterine and postnatal mortality are less understood and currently under investigation. Therefore, it is advisable to consistently conduct postmortem examinations in such cases of death.

Toxicological evaluation performed on fetal or neonatal tissue is highly complex, given the limited amount of material and the unique metabolism of substances by organs that are still in the developmental stage. For drug pharmacogenetics, the rapidly evolving ontogenetic changes in the expression of proteins or processes linked to the maturation of neonatal organs determine an additional layer of complexity beyond genetic variability, involving polymorphisms in the genes responsible.

The findings from clinical, autopsy, and toxicological analyses should always be integrated in the forensic assessment of casework, and this awaits further studies on pharmacogenetics and epigenetics able to provide more insight into how opioids influence the placental function and fetal development.

## Data Availability

No new data were created or analyzed in this study. Data sharing is not applicable to this article.

## Supplementary Materials

The following supporting information can be downloaded at: https://www.mdpi.com/article/10.3390/children11030278/s1, Table S1: Studies on opioid exposure during pregnancy; Table S2: Fetal and infant outcomes associated with maternal opioid use; Table S3: Forensic techniques for detecting and quantifying opioids in maternal and infant samples; Table S4: Genetic and epigenetic factors associated with opioid-related adverse outcomes.

## References

[1] Kaltenbach K., Berghella V., Finnegan L. (1998). Opioid dependence during pregnancy. Effects and management. Obstet. Gynecol. Clin. N. Am..

[2] Sithisarn T., Granger D.T., Bada H.S. (2012). Consequences of prenatal substance use. Int. J. Adolesc. Med. Health.

[3] National Institute on Drug Abuse, Substance Use While Pregnant and Breast Feeding. https://nida.nih.gov/publications/research-reports/substance-use-in-women/substance-use-while-pregnant-breastfeeding.

[4] Substance Abuse and Mental Health Services Administration 2020 National Survey on Drug Use and Health: Detailed Tables. https://www.samhsa.gov/data/report/2020-nsduh-detailed-tables.

[5] Bertaso A., Gottardo R., Murari M., Mazzola M., Porpiglia N.M., Taus F., Beghini R., Gandini F., Bortolotti F. (2023). Hair testing applied to the assessment of in utero exposure to drugs: Critical analysis of 51 cases of the University Hospital of Verona. Drug Test. Anal..

[6] Friguls B., Joya X., Garcia-Serra J., Gómez-Culebras M., Pichini S., Martinez S., Vall O., Garcia-Algar O. (2012). Assessment of exposure to drugs of abuse during pregnancy by hair analysis in a Mediterranean island. Addiction.

[7] NIDA Heroin DrugFacts. https://nida.nih.gov/publications/drugfacts/heroin.

[8] Huestis M.A., Choo R.E. (2002). Drug abuse’s smallest victims: In utero drug exposure. Forensic Sci. Int..

[9] Lam S.K., To W.K., Duthie S.J., Ma H.K. (1992). Narcotic addiction in pregnancy with adverse maternal and perinatal outcome. Aust. N. Z. J. Obstet. Gynaecol..

[10] British Columbia Centre on Substance Use A Guideline for the Clinical Management of Opioid Use Disorder—Pregnancy Supplement. https://www.bccsu.ca/wp-content/uploads/2018/06/OUD-Pregnancy.pdf.

[11] Fishman B., Daniel S., Koren G., Lunenfeld E., Levy A. (2019). Pregnancy outcome following opioid exposure: A cohort study. PLoS ONE.

[12] Pötsch L., Skopp G., Emmerich T.P., Becker J., Ogbuhui S. (1999). Report on intrauterine drug exposure during second trimester of pregnancy in a heroin-associated death. Ther. Drug Monit..

[13] Winklbaur B., Kopf N., Ebner N., Jung E., Thau K., Fischer G. (2008). Treating pregnant women dependent on opioids is not the same as treating pregnancy and opioid dependence: A knowledge synthesis for better treatment for women and neonates. Addiction.

[14] Rausgaard N.L.K., Broe A., Bliddal M., Nohr E.A., Ibsen I.O., Albertsen T.L., Ravn P., Damkier P. (2023). Use of opioids among pregnant women 1997-2016: A Danish drug utilization study. Eur. J. Obstet. Gynecol. Reprod. Biol..

[15] Bhatt-Mehta V., Jing X., Wang X., Zhu H.J. (2023). Transplacental methadone exposure and risk of Neonatal Opioid Withdrawal Syndrome. Pharmacotherapy.

[16] Bashore R.A., Ketchum J.S., Staischm K.J., Barrett C.T., Zimmermann E.G. (1981). Heroin addiction and pregnancy. West. J. Med..

[17] Anbalagan S., Mendez M.D. (2023). Neonatal Abstinence Syndrome.

[18] Ryan K.S., Prewitt K.C., Hayer S., Hedges M.A., Benson A.E., Lo J.O. (2023). Opioid Use in Pregnancy: A Review. Obstet. Gynecol. Surv..

[19] Esposito D.B., Bateman B., Werler M., Straub L., Mogun H., Hernandez-Diaz S., Huybrechts K. (2022). Ischemic Placental Disease, Preterm Delivery, and Their Association With Opioid Use During Pregnancy. Am. J. Epidemiol..

[20] Borrelli K.N., Wachman E.M., Beierle J.A., Taglauer E.S., Jain M., Bryant C.D., Zhang H. (2022). Effect of Prenatal Opioid Exposure on the Human Placental Methylome. Biomedicines.

[21] Little B., Snell L.M., Klein V.R., Gilstrap L.C., Knoll K.A., Breckenridge J.D. (1990). Maternal and fetal effects of heroin addiction during pregnancy. J. Reprod. Med..

[22] O’Donnell F.T., Jackson D.L. (2017). Opioid Use Disorder and Pregnancy. Mo. Med..

[23] Kandall S.R., Albin S., Lowinson J., Berle B., Eidelman A.I., Gartner L.M. (1976). Differential effects of maternal heroin and methadone use on birthweight. Pediatrics.

[24] Wolff K., Perez-Montejano R. (2014). Opioid neonatal abstinence syndrome: Controversies and implications for practice. Curr. Drug Abus. Rev..

[25] Athanasakis E., Karavasiliadou S., Styliadis I. (2011). The factors contributing to the risk of sudden infant death syndrome. Hippokratia.

[26] Maguire D.J., Taylor S., Armstrong K., Shaffer-Hudkins E., Germain A.M., Brooks S.S., Cline G.J., Andersen J.M., Høiseth G., Nygaard E. (2020). Prenatal exposure to methadone or buprenorphine and long-term outcomes: A meta-analysis. Early Hum. Dev..

[27] Benck K.N., Seide K., Jones A.K., Omori M., Rubinstein L.B., Beckwith C., Nowotny K.M. (2023). United States county jail treatment and care of pregnant incarcerated persons with opioid use disorder. Drug Alcohol Depend..

[28] Burns L., Conroy E., Mattick R.P. (2010). Infant mortality among women on a methadone program during pregnancy. Drug Alcohol Rev..

[29] Caritis S.N., Panigrahy A. (2019). Opioids affect the fetal brain: Reframing the detoxification debate. Am. J. Obstet. Gynecol..

[30] Cohen M.C., Morley S.R., Coombs R.C. (2015). Maternal use of methadone and risk of sudden neonatal death. Acta Paediatr..

[31] Conradt E., Flannery T., Aschner J.L., Annett R.D., Croen L.A., Duarte C.S., Friedman A.M., Guille C., Hedderson M.M., Hofheimer J.A. (2019). Prenatal Opioid Exposure: Neurodevelopmental Consequences and Future Research Priorities. Pediatrics.

[32] Concheiro M., Lendoiro E., de Castro A., Gónzalez-Colmenero E., Concheiro-Guisan A., Peñas-Silva P., Macias-Cortiña M., Cruz-Landeira A., López-Rivadulla M. (2017). Bioanalysis for cocaine, opiates, methadone, and amphetamines exposure detection during pregnancy. Drug. Test. Anal..

[33] de Castro A., Jones H.E., Johnson R.E., Gray T.R., Shakleya D.M., Huestis M.A. (2011). Methadone, cocaine, opiates, and metabolite disposition in umbilical cord and correlations to maternal methadone dose and neonatal outcomes. Ther. Drug. Monit..

[34] Dryden C., Young D., Hepburn M., Mactier H. (2009). Maternal methadone use in pregnancy: Factors associated with the development of neonatal abstinence syndrome and implications for healthcare resources. BJOG.

[35] Duska M., Goodman D. (2022). Implementation of a prenatal naloxone distribution program to decrease maternal mortality from opioid overdose. Matern. Child. Health J..

[36] Epstein R.A., Bobo W.V., Martin P.R., Morrow J.A., Wang W., Chandrasekhar R. (2013). Increasing pregnancy-related use of prescribed opioid analgesics. Ann. Epidemiol..

[37] Galli J., Loi E., Franzoni A., Accorsi P., Micheletti S., Pansera L., Fazzi E. (2023). Long-Term Visual and Neurodevelopmental Outcomes in Two Children with Congenital Nystagmus Secondary to Methadone Exposure In utero. Neuropediatrics.

[38] Garrison L., Leeman L., Savich R.D., Gutierrez H., Rayburn W.F., Bakhireva L.N. (2016). Fetal Growth Outcomes in a Cohort of Polydrug- and Opioid-Dependent Patients. J. Reprod. Med..

[39] Irnes E., Oltedal L., Bartsch H., Eide G.E., Elgen I.B., Aukland S.M. (2017). Brain morphology in school-aged children with prenatal opioid exposure: A structural MRI study. Early Hum. Dev..

[40] Kandall S.R., Gaines J. (1991). Maternal substance use and subsequent sudden infant death syndrome (SIDS) in offspring. Neurotoxicol. Teratol..

[41] Kandall S.R., Gaines J., Habel L., Davidson G., Jessop D. (1993). Relationship of maternal substance abuse to subsequent sudden infant death syndrome in offspring. J. Pediatr..

[42] Kushnir A., Bhavsar R., Hanna E., Hegyi T. (2023). Neonatal Abstinence Syndrome in Infants with Prenatal Exposure to Methadone versus Buprenorphine. Children.

[43] McCarthy J.J. (2023). Buprenorphine versus Methadone in Pregnancy. N. Engl. J. Med..

[44] López P., Bermejo A.M., Tabernero M.J., Cabarcos P., Alvarez I., Fernández P. (2009). Cocaine and opiates use in pregnancy: Detection of drugs in neonatal meconium and urine. J. Anal. Toxicol..

[45] McGlone L., Hamilton R., McCulloch D.L., MacKinnon J.R., Bradnam M., Mactier H. (2014). Visual outcome in infants born to drug-misusing mothers prescribed Methadone in pregnancy. Br. J. Ophthalmol..

[46] Monnelly V.J., Hamilton R., Chappell F.M., Mactier H., Boardman J.P. (2019). Childhood neurodevelopment after prescription of maintenance methadone for opioid dependency in pregnancy: A systematic review and meta-analysis. Dev. Med. Child. Neurol..

[47] Montanari E., Bonasoni M.P., Licata M., Salomone A., Gerace E., Vivarelli M., Giorgetti R., Tagliabracci A. (2018). Toxicological and histological analyses for a stillborn delivered by a mother under methadone maintenance therapy. Forensic Toxicol..

[48] Montanari E., Bonasoni M.P., Alessandrini F., Frazzi R., Mocchegiani F., Busardò F.P., Giorgetti R., Tagliabracci A. (2019). CYP2B6, ABCB1 and OPRM1 profile in a stillborn affected by chronic methadone intoxication. Forensic Toxicol..

[49] Newbury J., Sargayoos M., Bora S., Henderson J. (2023). Associations between social adversity, caregiver psychological factors, and language outcomes in 9.5-year-old children born to women with opioid use disorder. Child. Neuropsychol..

[50] Pandya V., Wilker C., Johnson-Davis K.L. (2023). Longitudinal trends in meconium drug detection in 46 US states between the years 2015 and 2020. J. Anal. Toxicol..

[51] Ordean A., Tubman-Broeren M. (2023). Safety and Efficacy of Buprenorphine-Naloxone in Pregnancy: A Systematic Review of the Literature. Pathophysiology.

[52] Ostrea E.M., Knapp D.K., Tannenbaum L., Ostrea A.R., Romero A., Salari V. (2001). Estimates of illicit drug use during pregnancy by maternal interview, hair analysis, and meconium analysis. J. Pediatr..

[53] Parikh R., Hussain T., Holder G., Bhoyar A., Ewer A.K. (2011). Maternal methadone therapy increases QTc interval in newborn infants. Arch. Dis. Child Fetal Neonatal Ed..

[54] Ramirez-Cacho W.A., Flores S., Schrader R.M., McKay J., Rayburn W.F. (2006). Effect of chronic maternal methadone therapy on intrapartum fetal heart rate patterns. J. Soc. Gynecol. Investig..

[55] Robert S., Goodwin D., Wilkins G.O., Averin R., Choo E., Schroeder J.R., Jasinski D.R., Rolley E.J., Hendrée E.J., Huestis M.A. (2007). Buprenorphine and Norbuprenorphine in Hair of Pregnant Women and Their Infants after Controlled Buprenorphine Administration. Clin. Chem..

[56] Ross E., Graham D., Money K. (2015). Developmental Consequences of Fetal Exposure to Drugs: What We Know and What We Still Must Learn. Neuropsychopharmacology.

[57] Serra A.E., Lemon L.S., Mokhtari N.B., Parks W.T., Catov J.M., Venkataramanan R., Caritis S.N. (2017). Delayed villous maturation in term placentas exposed to opioid maintenance therapy: A retrospective cohort study. Am. J. Obstet. Gynecol..

[58] Spowart K.M., Reilly K., Mactier H., Hamilton R. (2023). Executive functioning, behavioural, emotional, and cognitive difficulties in school-aged children prenatally exposed to methadone. Front. Pediatr..

[59] Ward S.L., Bautista D., Chan L., Derry M., Lisbin A., Durfee M.J., Mills K.S., Keens T.G. (1990). Sudden infant death syndrome in infants of substance-abusing mothers. J. Pediatr..

[60] Towers C.V., Hyatt B.W., Visconti K.C., Chernicky L., Chattin K., Fortner K.B. (2019). Neonatal head circumference in newborns with neonatal abstinence syndrome. Pediatrics.

[61] Whiteman V.E., Salemi J.L., Mogos M.F., Cain M.A., Aliyu M.H., Salihu H.M. (2014). Maternal opioid drug use during pregnancy and its impact on perinatal morbidity, mortality, and the costs of medical care in the United States. J. Pregnancy.

[62] Walhovd K.B., Westlye L.T., Moe V. (2010). White matter characteristics and cognition in prenatally opiate- and polysubstance-exposed children: A diffusion tensor imaging study. Am. J. Neuroradiol..

[63] Wurst K.E., Zedler B.K., Joyce A.R., Sasinowski M., Murrelle E.L. (2016). A Swedish Population-based Study of Adverse Birth Outcomes among Pregnant Women Treated with Buprenorphine or methadone: Preliminary Findings. Subst. Abus..

[64] Zedler B.K., Mann A.L., Kim M.M., Amick H.R., Joyce A.R., Murrelle E.L., Jones H.E. (2016). Buprenorphine compared with methadone to treat pregnant women with opioid use disorder: A systematic review and meta-analysis of safety in the mother, fetus and child. Addiction.

[65] Zipursky J., Juurlink D.N. (2021). Opioid use in pregnancy: An emerging health crisis. Obstet. Med..

[66] Crews K.R., Monte A.A., Huddart R., Caudle K.E., Kharasch E.D., Gaedigk A., Dunnenberger H.M., Leeder J.S., Callaghan J.T., Samer C.F. (2021). Clinical Pharmacogenetics Implementation Consortium Guideline for CYP2D6, OPRM1, and COMT Genotypes and Select Opioid Therapy. Clin. Pharmacol. Ther..

[67] CPIC Guideline for Opioids and CYP2D6, OPRM1, and COMT. https://cpicpgx.org/guidelines/guideline-for-codeine-and-cyp2d6/.

[68] Yalçin N., Flint R.B., van Schaik R.H.N., Simons S.H.P., Allegaert K. (2022). The Impact of Pharmacogenetics on Pharmacokinetics and Pharmacodynamics in Neonates and Infants: A Systematic Review. Pharmgenom. Pers. Med..

[69] Madadi P., Avard D., Koren G. (2012). Pharmacogenetics of opioids for the treatment of acute maternal pain during pregnancy and lactation. Curr. Drug Metab..

[70] Mactier H., McLaughlin P., Gillis C., Osselton M.D. (2017). Variations in Infant CYP2B6 Genotype Associated with the Need for Pharmacological Treatment for Neonatal Abstinence Syndrome in Infants of Methadone-Maintained Opioid-Dependent Mothers. Am. J. Perinatol..

[71] McPhail B.T., Emoto C., Butler D., Fukuda T., Akinbi H., Vinks A.A. (2021). Opioid Treatment for Neonatal Opioid Withdrawal Syndrome: Current Challenges and Future Approaches. J. Clin. Pharmacol..

[72] Liu T., Lewis T., Gauda E., Gobburu J., Ivaturi V. (2016). Mechanistic population pharmacokinetics of morphine in neonates with abstinence syndrome after oral administration of diluted tincture of opium. J. Clin. Pharmacol..

[73] van Hoogdalem M.W., McPhail B.T., Hahn D., Wexelblatt S.L., Akinbi H.T., Vinks A.A., Mizuno T. (2021). Pharmacotherapy of neonatal opioid withdrawal syndrome: A review of pharmacokinetics and pharmacodynamics. Expert. Opin. Drug Metab. Toxicol..

[74] Wachman E.M. (2013). Association of OPRM1 and COMT single-nucleotide polymorphisms with hospital length of stay and treatment of neonatal abstinence syndrome. JAMA.

[75] Albano G.D., La Spina C., Pitingaro W., Milazzo V., Triolo V., Argo A., Malta G., Zerbo S. (2023). Intrauterine and Neonatal Exposure to Opioids: Toxicological, Clinical, and Medico-Legal Issues. Toxics.

[76] Kintz P. (2015). Contribution of in utero drug exposure when interpreting hair results in young children. Forensic Sci. Int..

[77] Koren G., Cairns J., Chitayat D., Gaedigk A., Leeder S.J. (2006). Pharmacogenetics of morphine poisoning in a breastfed neonate of a codeine-prescribed mother. Lancet.

[78] Madadi P., Ross C.J., Hayden M.R., Carleton B.C., Gaedigk A., Leeder J.S., Koren G. (2009). Pharmacogenetics of neonatal opioid toxicity following maternal use of codeine during breastfeeding: A case-control study. Clin. Pharmacol. Ther..

[79] Friedrichsdorf S.J., Nugent A.P., Strobl A.Q. (2013). Codeine-associated pediatric deaths despite using recommended dosing guidelines: Three case reports. J. Opioid. Manag..

[80] Madadi P. (2007). Safety of codeine during breastfeeding: Fatal morphine poisoning in the breastfed neonate of a mother prescribed codeine. Can. Fam. Physician.

[81] Sistonen J. (2012). Prediction of codeine toxicity in infants and their mothers using a novel combination of maternal genetic markers. Clin. Pharmacol. Ther..

[82] Claessens A.J., Risler L.J., Eyal S., Shen D.D., Easterling T.R., Hebert M.F. (2010). CYP2D6 mediates 4-hydroxylation of clonidine in vitro: Implication for pregnancy-induced changes in clonidine clearance. Drug Metab. Dispos..

[83] Badaoui S., Hopkins A.M., Rodrigues A.D., Miners J.O., Sorich M.J., Rowland A. (2021). Application of Model Informed Precision Dosing to Address the Impact of Pregnancy Stage and CYP2D6 Phenotype on Foetal Morphine Exposure. AAPS J..

[84] Pogliani L., Mameli C., Cattaneo D., Clementi E., Meneghin F., Radice S., Bruno S., Zuccotti G.V. (2012). Acute kidney injury in a preterm infant homozygous for the C3435T polymorphism in the ABCB1 gene given oral morphine. Clin. Kidney J..

[85] Elens L., Norman E., Matic M., Rane A., Fellman V., van Schaik R.H. (2016). Genetic Predisposition to Poor Opioid Response in Preterm Infants: Impact of KCNJ6 and COMT Polymorphisms on Pain Relief After Endotracheal Intubation. Ther. Drug Monit..

[86] Matic M., Simons S.H., A van Lingen R., van Rosmalen J., Elens L., de Wildt S.N., Tibboel D., van Schaik R.H., Xie S., Ma W. (2014). Rescue morphine in mechanically ventilated newborns associated with combined OPRM1 and COMT genotype. Pharmacogenomics.

[87] Hronová K., Pokorná P., Posch L., Slanař O. (2016). Sufentanil and midazolam dosing and pharmacogenetic factors in pediatric analgosedation and withdrawal syndrome. Physiol. Res..

[88] Matic M., Norman E., Rane A., Beck O., Andersson M., Elens L., Tibboel D., Fellman V., van Schaik R.H. (2014). Effect of UGT2B7 −900G>A (−842G>A; rs7438135) on morphine glucuronidation in preterm newborns: Results from a pilot cohort. Pharmacogenomics.

[89] Matic M., de Wildt S.N., Elens L., de Hoon J.N., Annaert P., Tibboel D., van Schaik R.H., Allegaert K. (2016). SLC22A1/OCT1 Genotype Affects O-desmethyltramadol Exposure in Newborn Infants. Ther. Drug Monit..

[90] Baldo B.A. (2023). Neonatal opioid toxicity: Opioid withdrawal (abstinence) syndrome with emphasis on pharmacogenomics and respiratory depression. Arch. Toxicol..

[91] Dennis B.B., Bawor M., Thabane L. (2014). Impact of ABCB1 and CYP2B6 genetic polymorphisms on methadone metabolism, dose and treatment response in patients with opioid addiction: A systematic review and meta-analysis. PLoS ONE.

[92] Wachman E.M., Hayes M.J., Lester B.M. (2014). Epigenetic variation in the mu-opioid receptor gene in infants with neonatal abstinence syndrome. J. Pediatr..

[93] Wachman E.M., Hayes M.J., Shreatha J. (2018). Epigenetic variation in OPRM1 gene in opioid-exposed mother-infant dyads. Genes Brain Behav..

[94] Committee Opinion No (2017). Opioid Use and Opioid Use Disorder in Pregnancy. Obstet. Gynecol..

[95] Cook J.L. (2022). Epidemiology of opioid use in pregnancy. Best Pract. Res Clin. Obstet. Gynaecol..

[96] Martin C.E., Shadowen C., Thakkar B., Oakes T., Gal T.S., Moeller F.G. (2020). Buprenorphine dosing for the treatment of opioid use disorder through pregnancy and postpartum. Curr. Treat. Options Psychiatry.

[97] Sanjanwala A.R., Lim G., Krans E.E. (2023). Opioids and Opioid Use Disorder in Pregnancy. Obstet. Gynecol. Clin. N. Am..

[98] Smid M.C., Stone N.M., Baksh L., Debbink M.P., Einerson B.D., Varner M.W., Gordon A.J., Clark E.A.S. (2019). Pregnancy-Associated Death in Utah: Contribution of Drug-Induced Deaths. Obstet. Gynecol..

[99] Lavergne J., Langman E., Mansell D., Dol J., West C., Benoit B. (2023). Procedural pain assessment in neonates at risk of neonatal opioid withdrawal syndrome: A scoping review protocol. JBI Evid. Synth..

[100] Vella A., Savona-Ventura C., Mahmood T. (2023). Harmful effects of opioid use in pregnancy: A scientific review commissioned by the European Board and College of obstetrics and gynaecology (EBCOG). Eur. J. Obstet. Gynecol. Reprod. Biol..

[101] Ahmed N., Kassis A., Malone J., Yang J., Zamzami E., Lin A.H., Gordon S.M., Gong M.C., Bardo M., Dalmasso C. (2023). Prenatal Morphine Exposure Increases Cardiovascular Disease Risk and Programs Neurogenic Hypertension in the Adult Offspring. Hypertension.

[102] Alaee E., Pachenari N., Khani F., Semnanian S., Shojaei A., Azizi H. (2023). Enhancement of neuronal excitability in the medial prefrontal cortex following prenatal morphine exposure. Brain Res. Bull..

[103] Jullien S. (2021). Sudden infant death syndrome prevention. BMC Pediatr..

[104] Goldberg N., Rodriguez-Prado Y., Tillery R., Chua C. (2018). Sudden Infant Death Syndrome: A Review. Pediatr. Ann..

[105] Van Niekerk C., Van Deventer B.S., du Toit-Prinsloo L. (2017). Long QT syndrome and sudden unexpected infant death. J. Clin. Pathol..

[106] Ioakeimidis N.S., Papamitsou T., Meditskou S., Iakovidou-Kritsi Z. (2017). Sudden infant death syndrome due to long QT syndrome: A brief review of the genetic substrate and prevalence. J. Biol. Res..

[107] Hahn D., Emoto C., Euteneuer J.C., Mizuno T., Vinks A.A., Fukuda T. (2019). Influence of OCT1 Ontogeny and Genetic Variation on Morphine Disposition in Critically Ill Neonates: Lessons From PBPK Modeling and Clinical Study. Clin. Pharmacol. Ther..

[108] Willmann S., Edginton A.N., Coboeken K., Ahr G., Lippert J. (2009). Risk to the breast-fed neonate from codeine treatment to the mother: A quantitative mechanistic modeling study. Clin. Pharmacol. Ther..

[109] Beaulieu M.J. (2013). Oral clonidine in the management of acquired opioid dependency. Neonatal Netw..

[110] Stevens J.C., Marsh S.A., Zaya M.J. (2008). Developmental changes in human liver CYP2D6 expression. Drug Metab. Dispos..

[111] Hahn D., Fukuda T., Euteneuer J.C., Mizuno T., Vinks A.A., Sadhasivam S., Emoto C. (2020). Influence of MRP3 Genetics and Hepatic Expression Ontogeny for Morphine Disposition in Neonatal and Pediatric Patients. J. Clin. Pharmacol..

[112] Dobrinas M., Crettol S., Oneda B., Lahyani R., Rotger M., Choong E., Lubomirov R., Csajka C., Eap C.B. (2013). Contribution of CYP2B6 alleles in explaining extreme (S)- methadone plasma levels: A CYP2B6 gene resequencing study. Pharmacogenet. Genom..

[113] Gadel S., Crafford A., Regina K., Kharasch E.D. (2013). Methadone N-demethylation by the common CYP2B6 allelic variant CYP2B6.6. Drug Metab. Dispos..

[114] Kharasch E.D., Regina K.J., Blood J., Friedel C. (2015). Methadone pharmacogenetics: CYP2B6 polymorphisms determine plasma concentrations, clearance, and metabolism. Anesthesiology.

[115] Kringen M.K., Chalabianloo F., Bernard J.P., Bramness J., Molden E., Høiseth G. (2017). Combined effect of CYP2B6 genotype and other candidate genes on a steady-state serum concentration of methadone in opioid maintenance treatment. Ther. Drug Monit..

[116] Bunten H., Liang W.J., Pounder D.J., Seneviratne C., Osselton D. (2010). OPRM1 and CYP2B6 gene variants as risk factors in methadone -related deaths. Clin. Pharmacol. Ther..

[117] Bunten H., Liang W.J., Pounder D.J., Seneviratne C., Osselton D. (2011). Interindividual variability in the prevalence of OPRM1 and CYP2B6 gene variations may identify drug-susceptible populations. J. Anal. Toxicol..

[118] Nielsen D.A., Yuferov V., Hamon S. (2009). Increased OPRM1 DNA methylation in lymphocytes of methadone -maintained former heroin addicts. Neuropsychopharmacology.

[119] Vassoler F.M., Byrnes E.M., Pierce R.C. (2014). The impact of exposure to addictive drugs on future generations: Physiological and behavioral effects. Neuropharmacology.

