# Supplementary material for: Fetal and Infant Effects of Maternal Opioid Use during Pregnancy: A Literature Review including Clinical, Toxicological, Pharmacogenomic, and Epigenetic Aspects for Forensic Evaluation
Source: Children (Basel). 2024 Feb 23;11(3):278. doi: 10.3390/children11030278 (PMC10969201; doi:10.3390/children11030278)
Supplement: Supplementary file 1 [file children-11-00278-s001.zip › children-2827780-supplementary.pdf]

Table S1. Studies on opioid exposure during pregnancy.

Article	Article type	Opioid class	Sample size	Issue of interest	Main findings
Kaltenbach et al. [1]	Review	Metahdone, heroin	/	Foetus and infant cause of death	Overview of the main fetal and infant complications
Sithisarn et al. [2]	Review	Opiates	/	Foetus and infant cause of death	Overview of the main infant complications
Bertaso et al. [5]	Retrospective study	Morphine, methadone	51	Toxicological analyses	Assessment of in utero exposure to drugs
Friguls et al. [6]	Experimental study	Opiates	107	Foetus and infant cause of death	Assessment of in utero exposure to drugs
Lam et al. [9]	Retrospective study	Methadone, heroin	51	Foetus and infant cause of death	Overview of the main fetal and infant complications
Fishman et al. [11]	Retrospective study	Propoxyphene, codeine, tramadol, oxycodone, fentanyl	3003	Foetus and infant cause of death	Analysis for total major malformations and for spina bifida
Pötsch et al. [12]	Case report	Heroin metabolites, morphine	1	Toxicological analyses	All the fetal specimens were investigated
Rausgaard et al. [14]	Retrospective study	Codeine, tramadol, oxycodone, morphine	10878 pregnant women	Foetus and infant cause of death	Trend modifications in opioid prescriptions in pregnancy in the period 1997-2016 and intrauterine effects of exposition
Bhatt-Mehta et al. [15]	Retrospective study	Methadone	11 opioid-dependent mother–infant dyads	Toxicological analyses	Evaluate maternal and umbilical cord plasma concentrations of methadone and EDDP
Bashore et al. [16]	Review	Heroin	/	Foetus and infant cause of death	Overview of the main fetal and infant complications
Anbalagan et al. [17]	Review	Methadone, heroin, buprenorphine	/	Foetus and infant cause of death	Neonatal abstinence syndrome
Ryan et al. [18]	Review	Methadone, heroin, buprenorphine	/	Foetus and infant cause of death	Overview of the main fetal and infant complications
Esposito et al. [19]	Retrospective study	Prescription opioid in pregnancy (except opioid replacement therapy)	1,833,871 pregant women	Foetus and infant cause of death	Overview of the main fetal and infant complications
Borrelli et al. [20]	Experimental study	Methadone/Fentanil/Buprenop hine	40	Epigenetic analyses	Prenatal opioid exposure and placental epigenetic changes lead to abnormal fetal brain development and NOWS
Little et al. [21]	Research study	Heroin	24	Foetus and infant cause of death	Overview of the main fetal and infant complications
O'Donnell et al. [22]	Communicati on	Heroin	/	Foetus and infant clinical complications	Overview on management of opioid use disorder in pregnancy
Kandall et al. [23]	Retrospective study	Methadone, heroin	337 newborns	Foetus and infant cause of death	Birth weight analysis

Wolff et al. [24]	Communication	Heroin, methadone	/	Foetus and infant clinical complications	Opioid Neonatal Abstinence Syndrome
Athanasakis et al [25]	Review	Opiates	/	Foetus and infant cause of death	Contributing factors to SIDS
Maguire et al. [26]	Meta-analysis	Methadone, buprenorphine	/	Foetus and infant clinical complications	Long-term outcomes of the prenatal exposure
Benck et al. [27]	Retrospective study	Methadone, buprenorphine	371	Foetus and infant clinical complications	Care of pregnant incarcerated persons with opioid use disorder
Burns et al. [28]	Retrospective study	Methadone	675310 liveborn infants	Foetus and infant cause of death	Risk of in infant death in methadone maintenance therapy
Caritis et al. [29]	Communication	Morphine, methadone	/	Foetus and infant clinical complications	Implication on fetal brain
Cohen et al. [30]	Research article	Heroin, methadone	138 neonatal autopsies	Infant cause of death	SIDS association
Conradt et al. [31]	Review	Morphine, methadone, buprenorphine	/	Foetus and infant clinical complications	Neurodevelopmental consequences
Concheiro et al. [32]	Research study	Opiods, methadone	727 mother–infant dyads	Toxicological analyses	Maternal hair resulted as the most sensitive specimen to detect drug exposure during pregnancy
de Castro et al. [33]	Research study	Methadone	19	Toxicological analyses	Umbilical cord methadone concentrations were correlated to methadone doses
Dryden et al. [34]	Retrospective study	Methadone	450	Foetus and infant clinical complications	Development of neonatal abstinence syndrome
Duska et al. [35]	Retrospective study	Methadone, naloxone	12	Foetus and infant cause of death	Provide a framework for other Ob/Gyn clinics to use in implementing similar naloxone distribution programs
Epstein et al. [36]	Retrospective study	Morphine, methadone, buprenorphine	14448	Foetus and infant clinical complications	Prevalence of prescribed opioid analgesics among pregnant women from 1995 to 2009
Galli et al. [37]	Case series	Methadone	2	Foetus and infant clinical complications	Long-Term Visual and Neurodevelopmental Outcomes
Garrison et al. [38]	Retrospective study	Morphine, methadone	59	Foetus and infant clinical complications	Fetal Growth Outcomes
Irnes et al. [39]	Retrospective study	Morphine, methadone	16	Foetus and infant clinical complications	Brain morphology with magnetic resonance imaging
Kandall et al. [40]	Review	Heroin, methadone	/	Foetus and infant cause of death	Association between opiates and SIDS
Kandall et al. [41]	Retrospective study	Heroin, methadone	1760 SIDS cases analyzed	Foetus and infant cause of death	Association between opiates and SIDS
Kushnir et al. [42]	Retrospective study	Methadone, buprenorphine	290	Foetus and infant clinical complications	Different between prenatally exposed to methadone or buprenorphine

McCarthy et al. [43]	Communication	Methadone, buprenorphine	/	Foetus and infant clinical complications	Buprenorphine versus Methadone in Pregnancy
López et al. [44]	Case report	Codeine	1	Toxicological analyses	levels of drugs disappeared by the third day
McGlone et al. [45]	Retrospective study	Methadone	81	Foetus and infant clinical complications	Abnormal visual electrophysiology
Monnelly et al. [46]	Meta-analysis	Methadone	/	Foetus and infant clinical complications	Childhood neurodevelopment
Montanari et al. [47]	Case report	Methadone	1	Foetus and infant cause of death	Methadone contribution to IUFD
Montanari et al. [48]	Case report	Methadone	1	Foetus and infant cause of death	Genetic assessment of methadone fetal clearance
Newbury et al. [49]	Retrospective study	Methadone	80	Foetus and infant clinical complications	Social adversity, caregiver psychological factors, and language outcomes
Pandya et al. [50]	Retrospective study	Morphine	175000	Toxicological analyses	Assessment of the most common drug combination in meconium
Ordean et al. [51]	Retrospective study	Buprenorphine	/	Toxicological analyses	buprenorphine-naloxone is a safe and effective opioid agonist treatment for pregnant people
Ostrea et al. [52]	Research study	Opiates	58	Toxicological analyses	Estimates of illicit drug use during pregnancy by hair analysis and meconium analysis
Parikh et al. [53]	Research study	methadone	36	Toxicological analyses	Newborns exposed to methadone are at risk of cardiac rhythm disturbances
Ramirez et al. [54]	Retrospective study	Methadone	56	Foetus and infant clinical complications	Intrapartum fetal heart rate patterns
Robert et al. [55]	Research study	Buprenorphine	9 mothers<br>4 infants	Toxicological analyses	Significant positive association between concentrations of buprenorphine and norbuprenorphine in maternal hair and a trend for this association in infant hair
Ross et al. [56]	Review	Opiates	/	Toxicological analyses	Drugs effect on developing nervous system
Serra et al. [57]	Retrospective study	Methadone, buprenorphine	86 (buprenorphine) + 268 (methadone) exposed placentas	Foetus and infant cause of death	Placental histological examination
Spowart et al. [58]	Retrospective study	Methadone	153	Foetus and infant clinical complications	Executive functioning, behavioural, emotional, and cognitive difficulties
Ward et al. [59]	Retrospective study	Opiates	497 infants	Foetus and infant cause of death	Effects of opiates on the fetus and the infant

Towers et al. [60]	Retrospective study	Morphine, methadone	429	Foetus and infant clinical complications	Head circumference in neonatal abstinence syndrome
Whiteman et al. [61]	Retrospective study	Opioid	138,224 pregnant women	Foetus and infant cause of death	Effects of opiates on the fetus and the infant
Walhovd et al. [62]	Research study	opiates	14	Toxicological analyses	Myelin may be particularly vulnerable to prenatal substance exposure
Wurst et al. [63]	Research study	Buprenorphine, methadone	176 (buprenorphine) + 52 (methadone) pregnant women	Foetus and infant cause of death	Effects of opiates on the fetus and the infant
Zedler et al. [64]	Review	Buprenorphine, methadone	/	Toxicological analyses	Evidence of lower risk of preterm birth, greater birth weight and larger head circumference with buprenorphine treatment during pregnancy compared with methadone treatment
Zipursky et al. [65]	Review	Opioids	/	Toxicological analyses	Opioid use in pregnancy and how this relates to maternal and neonatal health outcomes
Crews et al. [66]	Review	Codeine, tramadol, hydrocodone, oxycodone, methadone	/	Pharmacogenetic analyses	Implementation Consortium Guidelines for opioids therapy and *CYP2D6*, *OPRM1,* and *COMT* genotyping
CPIC Guideline [67]	Guidelines	Drugs	/	Pharmacogenetic analyses	Pharmacogenetic test and drug therapy
Yalçin et al. [68]	Review	Morphine, methadone, tramadol, sufentanil, midazolam	/	Pharmacogenetic analyses	Pharmacogenetics in infants and neonates
Madadi et al. [69]	Review	Morphine, synthetic and semi-synthetic opioids	/	Pharmacogenetic analyses	Pharmacogenetics variants and pregnancy
McPhail et al. [71]	Review	Methadone, buprenorphine, morphine	/	Foetus and infant clinical complications	Pharmacokinetic and pharmacodynamic of opioids in infants with NOWS: challenges and future approaches
Liu et al. [72]	Clinical trial study	Morphine	34	Pharmacokinetic model analysis	Description of the population PK of morphine in NAS
van Hoogdalem et al. [73]	Review	Buprenorphine, clonidine, methadone, morphine	/	Pharmacogenetic analyses	Influence of gestational age, postnatal age, and pharmacogenetics on the pharmacokinetics of drug therapies used in NOWS.

Wachman et al. [74]	Multicenter cohort study	Methadone, buprenorphine	86	Pharmacogenetic analyses	OPRM1 and COMT gene variants were associated with a shorter length of hospital stay
Albano et al. [75]	Review	Opioids	/	Toxicological analyses	Evidence-based approach for managing neonatal exposure to opioids
Kintz et al. [76]	Research study	Heroin, methadone	24	Toxicological analyses	Interpretation issue in assessing neonatal drug exposure
Baldo et al. [90]	Review	Methadone, buprenorphine, morphine	/	Pharmacogenetic analyses	Pharmacogenomics of NOWS and respiratory depression
Wachman et al. [93]	Experimental study	Methadone, buprenorphine	86	Epigenetic analyses	Increased methylation within the OPRM1 promoter is associated with worse NAS outcomes

Table S2. Fetal and infant outcomes associated with maternal opioid use.

**Article**	**Opioid class**	**Outcomes**
Kaltenbach et al. [1]	Methadone, heroin	Spontaneous abortion, IUFD, IUGR, preeclampsia, premature labor/delivery, PROM, abruptio placentae, intrauterine passage of meconium, low Apgar scores
Sithisarn et al. [2]	Opiates	SGA, altered neurobehavioral in infancy
Huestis et al. [8]	Methadone, heroin	Increased risk of abortion in case of sudden withdrawal, IUGR, SGA, NOWS; increased risk of SIDS
Lam et al. [9]	Methadone, heroin	Prematurity, SGA, antepartum hemorrhage, NOWS
Fishman et al. [11]	Propoxyphene, codeine, tramadol, oxycodone, fentanyl	propoxyphene and codeine: perinatal death, low birth weight, SGA, low Apgar score (<8) at 1 and 5 minutes after birth; codeine: increased risk of spina bifida; third trimester exposition of opioids: low birth weight, perinatal death.
Rausgaard et al. [14]	Codeine, tramadol, oxycodone, morphine	No variations in prescription drugs and no difference in livebirth or stillborn babies
Bashore et al. [16]	Heroin	Preeclampsia, IUGR, IUFD, NOWS
Anbalagan et al. [17]	Methadone, heroin, buprenorphine	NOWS, neurodevelopmental delays, behavioral problems, death
Ryan et al. [18]	Methadone, heroin, buprenorphine	placental insufficiency, preterm birth, NOWS, low birth weight, SGA
Esposito et al. [19]	Prescription opioids in pregnancy (except opioid replacement therapy)	minimal increased risk of placental abruption, preterm birth, and SGA
Little et al. [21]	Heroin	Preterm birth, SGA, low birth weight infants
O'Donnell et al. [22]	Heroin	Neonates exposed to opioids in utero have a risk of neonatal abstinence syndrome (NAS) that is as high as 90%
Kandall et al. [23]	Methadone, heroin	IUGR, low birth weight

Wolff et al. [24]	Heroin, methadone	Generalized motor seizures and myoclonic jerks rates of occurrence ranging from 2% to 10% for infants withdrawing from opioids
Athanasakis et al [25]	Opiates	Pregnancy exposition contribute to SIDS
Maguire et al. [26]	Methadone, buprenorphine	Worse outcomes for impaired vision
Benck et al. [27]	Methadone, buprenorphine	Accessing medications prevent erratic fetal opioid level withdrawals, fetal and neonatal morbidity and mortality
Burns et al. [28]	Methadone	Higher infant death rate, mainly SIDS, in methadone maintenance therapy
Caritis et al. [29]	Morphine, methadone	Alterations in white matter of the centrum ovale, longitudinal fasciculi, and capsules
Cohen et al. [30]	Heroin, methadone	SIDS
Conradt et al. [31]	Morphine, methadone, buprenorphine	Alterations in IQ, neurologic performance and language performance
Dryden et al. [34]	Methadone	Smaller birthweight and head circumference
Epstein et al. [36]	Morphine, methadone, buprenorphine	Birth defects with early fetal exposure and neonatal abstinence syndrome with later fetal exposure
Galli et al. [37]	Methadone	In all cases ophthalmologic, oculomotor, and perceptive problems
Garrison et al. [38]	Morphine, methadone	49.2% incidence of microcephaly
Irnes et al. [39]	Morphine, methadone	6.5% smaller basal ganglia, 9.2% caudate, 7.6% thalamus and 10.3% cerebellar white matter
Kandall et al. [40]	Heroin, methadone	Weak association between SIDS and opiate use
Kandall et al. [41]	Heroin, methadone	Opiate contribution to SIDS cause
Kushnir et al. [42]	Methadone, buprenorphine	birth weight between groups (neonates in the Bup/Bup+ being significantly heavier than the Met/Met+ ones)
McCarthy et al. [43]	Methadone, buprenorphine	Buprenorphine is associated with a lower risk of adverse out-comes than methadone concerning behavioural, emotional and cognitive regulation
McGlone et al. [45]	Methadone	40% failed clinical visual assessment; relative risk of abnormal assessment was 5.1
Monnelly et al. [46]	Methadone	Mental Development Index weighted mean difference of children -4.3
Montanari et al. [47]	Methadone	IUFD high likely due to chronic fetal MTD intoxication, placental delayed villous maturation, and chorionic thrombosis
Montanari et al. [48]	Methadone	Abnormal genetic profile of *CYP2B6*, *ABCB1* and *OPRM1* reducing methadone clearance
Newbury et al. [49]	Methadone	At age 9.5 years lower language scores than the non-exposed children
Ramirez et al. [54]	Methadone	Severe variable or late decelerations during the second stage of labor (44.2%)
Serra et al. [57]	Methadone, buprenorphine	Increased incidence of delayed villous maturation in the exposed placentas
Spowart et al. [58]	Methadone	Children had birthweights 236 g lighter on average and had smaller birth OFC by an average of 0.8 cm
Ward et al. [59]	Opiates	SIDS and cleft palate
Towers et al. [60]	Morphine, methadone	Head circumference for was 33.04 cm (±1.9 cm)

Whiteman et al. [61]	Prescription opioid in pregnancy	Preterm labor, PROM, IUGR, IUFD
Wurst et al. [63]	Methadone, buprenorphine	Reduced risk of NOWS in buprenorphine, in methadone higher frequency of preterm birth and congenital malformations

Table S3. Forensic techniques for detecting and quantifying opioids in maternal and infant samples.

Article	Analytical techniques
Bertaso et al. [5]	LC-MS/MS
Pötsch et al. [12]	GC-MS
Concheiro et al. [32]	LC-MS/MS
de Castro et al. [33]	LC-MS/MS
López et al. [44]	GC-MS
Pandya et al. [50]	LC-MS/MS
Ordean et al. [51]	/
Ostrea et al. [52]	GC-MS
Parikh et al. [53]	/
Robert et al. [55]	LC-MS/MS
Ross et al. [56]	/
Walhovd et al. [62]	/
Zedler et al. [64]	/
Zipursky et al. [65]	/
Albano et al. [75]	/
Kintz et al. [76]	LC-MS/MS

Table S4. Genetic and epigenetic factors associated with opioid-related adverse outcomes.

**Article**	**Article type**	**Opioid class**	**Sample size**	**Issue of interest**	**Main findings**
Montanari et al [48]	Experimental research	Methadone	Stillborn (N=1)	Pharmacogenetic analyses	Association between CYP2B6 variant alleles and stillborn methadone intoxication
Mactier et al. [70]	Observational cohort study	Methadone	MMOD mother and their newborns (N=21)	Pharmacogenetic analyses	Higher frequency of homozygous allele at 516 and 785 regions of *CYP2B6*

					gene in treated infants leading to severity of NAS
Wachman et al. [74]	Cohort study	Morphine/ Buprenorphine	Mother/infant pairs (N=113)	Genetic analyses	Infants with the rs1799971 AG or GG genotypes experience shorter hospital stays for the treatment of NAS in comparison to those with the AA genotype.
Koren et al [77]<br>*Madadi, et al [78]*<br>*Friedrichsdorf, et al. [79]*<br>*Madadi, et al [80]*<br>*Sistonen, et al [81]*	Case and cohort studies	Morphine/ Methadone	Mother-infant pairs (Total N=188)	Pharmacogenetic analyses	High risk of opioid toxicity in breastfed infant associated with *CYP2D6* and *ABCB1* mother's genetic polymorphism
Claessens et al [82]	Experimental study	Clonidine	Pregnant women (N=17)	Pharmacogenetic analyses	Increase in clonidine oral clearance in pregnant women primarily mediated by *CYP2D6*
Badaoui et al [83]	Pharmacokinetic modelling	Codeine/Morphi ne	Pregnant females/foetus (N=250)	Pharmacogenetic analyses	Higher risk to foetal morphine exposure in the first trimester of pregnancy
Pogliani et al [84]	Case report	Morphine	Pre-term newborn (N=1)	Pharmacogenetic analyses	Opioid-induced urinary retention linked to a homozygous for the C3435T polymorphism in the *ABCB1* gene
Elens et al [85]	Experimental study	Remifentanil/M orphine	Pre-term newborns (N=34)	Pharmacogenetic analyses	Predisposition to a slow opioid response in preterm newborns carrying the KCNJ6 -1250G.A and the COMT c.472G.A alleles
Matic et al [86]	Cohort study	Morphine	Pre-term newborns (N=34)	Pharmacogenetic analyses	The combined *OPRM1/COMT* 'high-risk' genotype led to a significant association with the need for rescue
Hronová et al [87]	Retrospective study	Sufentanil	Newborns (N=30)	Pharmacogenetic analyses	COMT and ABCB1 polymorphism influence the sufentanil dosing
Matic et al [88]	Cohort study	Morphine	Pre-term newborns (N=15)	Pharmacogenetic analyses	The UGT2B7 -900G>A polymorphism significantly alters morphine pharmacokinetics in preterm infants.
Matic et al. [89]	Retrospective study	Methadone	Pre-term ill newborns and infants (N=50)	Pharmacogenetic analyses	Combination of *CYP2D6* and *SLC22A1/OCT1* polymorphism influence O-desmethyltramadol PK
Dennis et al [91]	Review	Methadone	/	Pharmacogenetic analyses	Methadone metabolism is significantly slower in *6 homozygous carriers
Wachman al. [92]	Cohort study	Methadone or buprenorphine	Infants treated for NOWS (N=68)	Epigenetic analyses	High levels of OPRM1 methylation at specific CpG sites influence the increased NAS severity
